# Polycystin-1 Inhibits Cell Proliferation through Phosphatase PP2A/B56*α*

**DOI:** 10.1155/2019/2582401

**Published:** 2019-09-19

**Authors:** Yan Tang, JungWoo Yang, Wang Zheng, Jingfeng Tang, Xing-Zhen Chen, Jianzheng Yang, Zuocheng Wang

**Affiliations:** ^1^Department of Oncology and Haematology, The Second Hospital, Jilin University, Changchun 130041, China; ^2^Membrane Protein Disease Research Group, Department of Physiology, Faculty of Medicine and Dentistry, University of Alberta, Edmonton T6G 2H7, Canada; ^3^National “111” Center for Cellular Regulation and Molecular Pharmaceutics, Hubei University of Technology, Wuhan 430086, China; ^4^Department of Radiotherapy, The Second Hospital, Jilin University, Changchun 130041, China

## Abstract

Autosomal dominant polycystic kidney disease (ADPKD) is associated with a number of cellular defects such as hyperproliferation, apoptosis, and dedifferentiation. Mutations in polycystin-1 (PC1) account for ∼85% of ADPKD. Here, we showed that wild-type (WT) or mutant PC1 composed of the last five transmembrane (TM) domains and the C-terminus (termed PC1-5TMC) inhibits cell proliferation and protein translation, as well as the downstream effectors of mTOR, consistent with previous reports. Knockdown of B56*α*, a subunit of the protein phosphatase 2A (PP2A) complex, or application of PP2A inhibitor okadaic acid or calyculin A, abolished the inhibitory effect of PC1 and PC1-5TMC on proliferation, indicating that PP2A/B56*α* mediates the regulation of cell proliferation by PC1. In addition to the phosphorylated S6 and 4EBP1, B56*α* was also downregulated by PC1 and PC1-5TMC. Furthermore, the downregulation of B56*α*, which may be mediated by mTOR but not AKT, can account for the dependence of PC1-inhibited proliferation on PP2A.

## 1. Introduction

Autosomal dominant polycystic kidney disease (ADPKD) results from mutations in genes coding for polycystin 1 (PC1) and polycystin 2 (PC2), which account for about 85% and 15% of ADPKD, respectively [1]. Both loss and gain of PC1 or PC2 function result in cystogenesis [2]. PC1 is a 4302-amino acid (aa), 462-kDa protein containing 11 putative transmembrane (TM) spans. Its large extracellular N-terminus contains a number of motifs that suggest interaction with extracellular ligands and matrix proteins. The short C-terminus contains domains for G-protein activation and interaction with partner proteins. Thus, PC1 seems to function as a cell surface receptor that couples extracellular stimuli to intracellular signaling [3].

ADPKD is associated with several cellular abnormalities, including increased cell proliferation, apoptosis, and dedifferentiation. In addition to decreased Ca^2+^ signaling, several cell fate-related pathways are modulated by PC1/PC2, including cAMP, MAPK, Wnt, JAK-STAT, Hippo, Src, and mTOR [4]. Overexpression of PC1 or PC2 in renal epithelial cells was shown to repress cell proliferation through the JAK-STAT signaling pathway [5]. PC1 was found to function as a modulator of noncapacitive Ca^2+^ entry (NCCE) and Ca^2+^ oscillation, through which it affects cell proliferation [6]. PC1 was also found to regulate proliferation in PKD1-depleted or -mutated epithelial cells through a CREB-AP1 pathway which upregulates the amphiregulin level [7]. Moreover, the mammalian target of rapamycin (mTOR) pathway was shown to be abnormally activated in cyst-lining epithelial cells in human ADPKD patients and mouse models, which may result from loss of PC1 binding with tuberin, suggesting that PC1 inhibits cell proliferation by downregulating the activity of mTOR and its interaction with tuberin [8]. It was later verified PC1 reduces cell size by negatively regulating mTOR and downstream effectors S6K1 and 4EBP1 in a tuberin-dependent manner [9]. These studies together demonstrated that PC1 downregulates proliferation through different pathways and indicated that abnormalities of any of these pathways associated with ADPKD may account for the altered proliferation.

Protein-serine/threonine phosphatase 2A (PP2A) together with protein-serine/threonine phosphatase (PP1), PP2B, and PP4 to PP7 belongs to the phosphoprotein phosphatase family. Cellular PP2A exists in two forms, a core enzyme and a heterotrimeric holoenzyme [10]. Each PP2A holoenzyme is formed by a combination of three subunits: a common catalytic subunit PP2Ac containing the active site, a variable regulatory (B) subunit PP2Ab, and a structural A subunit PP2Aa. The function of PP2A relies on the B subunit that determines the substrate specificity and the subcellular localization of the PP2A complex [11]. The PP2A activity and subcellular localization are also regulated by posttranslational modifications of B56 that have homologous B56*α* and B56*β* isoforms subunit. Comparing with B56*β*, B56*α* is more highly expressed and has been widely studied [12]. PP2A is known to dephosphorylate over 300 types of substrates involved in almost all major cellular signaling pathways including the Wnt, mTOR, and MAPK pathways [13].

Phosphorylation and dephosphorylation are two opposite processes that control the activity of numerous cellular events. The activation of p70^s6k^ (also called 70-kDa S6 kinase) leads to an increase in the protein synthesis and cell proliferation through acting on its target substrate, the S6 ribosomal protein. Eukaryotic translation initiation factor 4E- (eIF4E-) binding protein 1 (4EBP1) is phosphorylated, resulting in its dissociation from eIF4E and activation of cap-dependent mRNA translation [14]. Because S6 and 4EBP1 are the best known substrates of mTOR [15], while PP2A regulates translation initiation through dephosphorylating 4EBP1 and p70^s6k^ [16], we speculate that PP2A is a major mTOR phosphatase to regulate downstream effectors. In this study, we employed culture cells transfected with PC1 or PC1 mutants to investigate the relationship between PC1-regulating cell proliferation/translation and PP2A (B56*α*).

## 2. Materials and Methods

### 2.1. Antibodies and Reagents

P-S6 and S6 were products of Cell Signaling Technology (NewEngland Biolabs, Pickering, ON). PP2A-B56*α*, 4EBP1, P-4EBP1, GFP (B-2), anti-*β*-actin, anti-Flag antibodies, and secondary antibodies were from Santa Cruz (Santa Cruz, Santa Cruz, CA). The PP2A inhibitor okadaic acid (OA) was from Calbiochem (EMD Chemicals Inc., Gibbstown, NJ); another PP2A inhibitor calyculin A (CA) was from Cell Signaling Technology (New England Bio labs). Rapamycin and puromycin were products of Sigma-Aldrich, Canada.

### 2.2. DNA Constructs, Cell Culture, and Transfection

Plasmid pcDNA3-GFP-PC1-5TMC (PC1-5TMC) comprising C-terminus of PC1 and last 5 transmembrane (TM) was constructed as described previously [17]. HEK293T, HeLa, IMCD3, and MDCK cells were grown in Dulbecco's Modified Eagle Medium (DMEM) with 10% fetal bovine serum, L-glutamine, and penicillin-streptomycin in 5% CO_2_ and 37°C. HEK293T cells stably being transfected with mouse full-length PC1 was from one co-author Dr. J. Yang and was grown with 2* μ*g/ml of puromycin under above conditions [18]. Transient transfection was carried out employing lipofectamine 2000 (Invitrogen, Burlington, ON).

### 2.3. Cell Proliferation Assay

HEK293T, HeLa, MDCK, or IMCD cells were transiently transfected in 100 mm dishes. At 24 hours (hr) after transfection, cell proliferation assay was performed as described previously [19, 20]. Cells were split and seeded into either separate wells of a 96-well plate labelling with or without OA or CA treatment or new 100 mm dishes for further knockdown experiments. After incubation for another 24 hr, luminescence activity was measured using Alarma-Blue (Invitrogen, Canada, Inc.) in serum-free medium and a microplate reader. HEK293T cells with stable transfection of wild-type (WT) mouse PC1 after 24 hr of culture revealed by WB were split and seeded into 96-well plates. The rest of cells in the 100 mm dishes were collected for immunoblotting at the same time point. The cell proliferation rate was calculated using the following formula: cell proliferation rate (%) = OD_test_/OD_control_ × 100%.

### 2.4. ^35^S Pulse Labelling

At 40 hr after transfection of HEK293T or HeLa cells, the cells were starved for 1 hr in L-methionine- and L-cysteine-free DMEM and then labelled with 50 *μ*Ci of (^35^S) methionine/cysteine (EXPRE ^35^S Protein Labeling Mix, PerkinElmer, Woodbridge, ON) for 10 minutes, and the cell extracts were employed for sodium dodecyl sulfate-polyacrylamide gel electrophoresis and autoradiography, as described previously [19].

### 2.5. RNA Interference

PP2A-B56*α* siRNA (Santa Cruz, Cat#: sc-39181) was used to interfere with the expression of B56*α* protein in HEK293T cells following product instruction. The efficiency of siRNA gene knockout was evaluated by blotting.

### 2.6. Statistics

Densities of bands were quantified by Image J software. Values generated were presented as mean ± standard error (SEM). *N* represents the number of independent experiments. Data were statistically analyzed by Sigma Plot 12 soft ware program (Systat Software Inc., San Jose, CA). The paired *t*-test is used to determine the difference in data between two groups. *P* value less than 0.05 was considered statistically significant.

## 3. Results

### 3.1. PC1 Inhibits Proliferation/Translation and Downstream Effectors of mTOR

**Figure 1:**
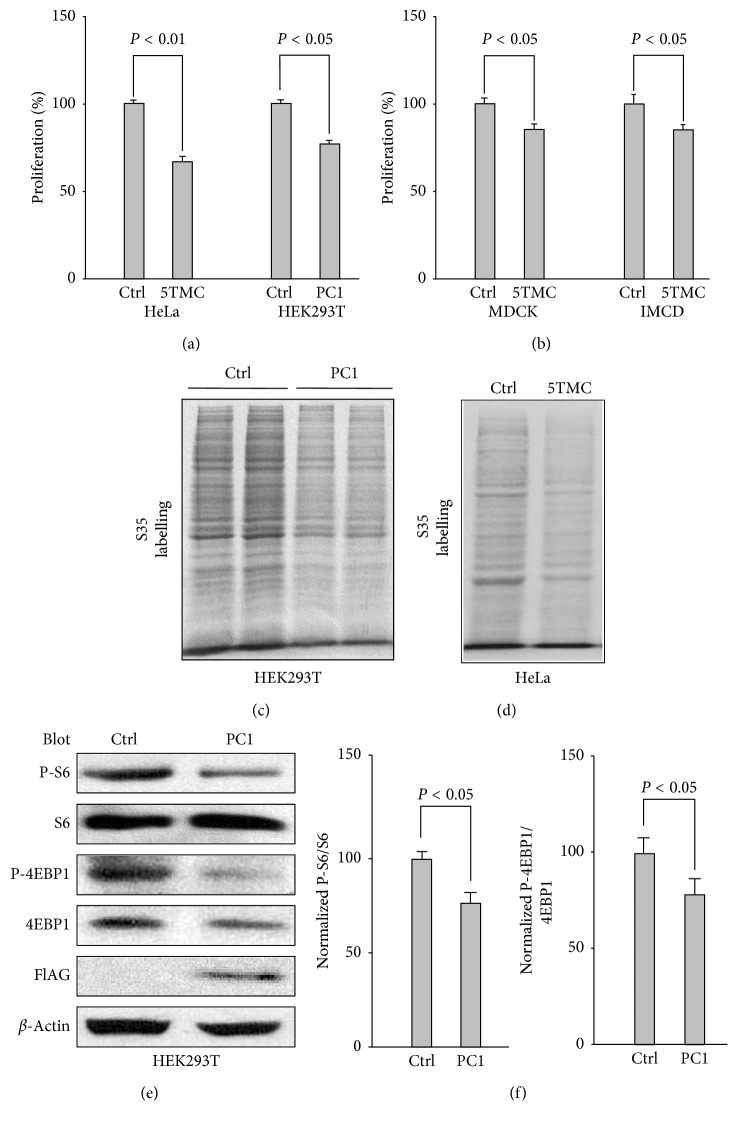
Downregulation of cell proliferation/protein translation and downstream effectors of mTOR by PC1. (a) Effects of PC1-5TMC or full-length PC1 on proliferation of HeLa and HEK293T cells. HeLa cells transiently expressing GFP-PC1-5TMC, GFP vector, and HEK293T stably expressing full-length mouse PC1 were plated in multiple wells of a 96-well plate and grown for another 24 hr for cell proliferation assay. Cell proliferation rate of control was normalized to 100%. Control (Ctrl), GFP vector; 5TMC, GFP-tagged PC1-5TMC; PC1, HEK293T cells stably expressing full-length PC1. Shown are averaged data (*N* = 4). (b) Effects of PC1-5TMC on proliferation of MDCK and IMCD3 cells. MDCK and IMCD3 cells replacing the above cells were transiently transfected with PC1-5TMC and GFP vector, and cell proliferation assay was performed as shown in (a) (*N* = 4). (c) Effects of full-length PC1 on protein synthesis. HEK293T cells stably expressing full-length PC1 were starved for 1 hr and then followed by pulse labelling for ^35^S pulse labelling assay. (d) Effects of PC1-5TMC in HeLa cells on protein synthesis. ^35^S pulse labelling assay of HeLa cells transiently expressing PC1-5TMC or GFP was performed as that of the above HEK293T cells in (c). (e) Effects of full-length PC1 on P-S6 and P-4EBP1. 60 *μ*g of total protein from HEK293T cells stably expressing flag-tagged WT PC1 or GFP vector was used for WB with the indicated antibodies. *β*-Actin served as loading controls. (f) Statistical data showing averaged and normalized ratios (%) of P-S6/S6 (*N* = 4) and P-4EBP1/4EBP1 (*N* = 4) from (e).

In order to clarify the effect of PC1 on cell proliferation, we used Alarma-Blue to label HeLa, human embryonic kidney (HEK293T), Madin-Darby canine kidney (MDCK), and mouse inner medullary collecting duct (IMCD3) cells after transfection of PC1-5TMC, a truncation mutant consisting of the last five TM domains (S7-S11), and the C-terminus of human PC1 and found that PC1-5TMC in HeLa, MDCK, and IMCD cells, as well as WT PC1 in HEK293T cells, suppresses proliferation (Figures 1(a) and 1(b)), consistent with previous reports [4–9]. The difference in magnitude of the responses can be explained mainly by the fact that different cell types can lead to different cell transfection efficiencies and thus different inhibition rates of cell proliferation.

Protein translation plays a critical role in the regulation of cellular processes associated with cell volume control and cell proliferation. We next utilized ^35^S labelling to determine whether PC1 modulates protein translation. We found that stably expressed WT PC1 and transiently expressed PC1-5TMC reduce protein synthesis in HEK293T and HeLa cells, respectively (Figures 1(c) and 1(d)). Therefore, PC1 repressed both the cell proliferation and mRNA translation.

PC1 is known to repress cell growth by downregulating mTOR and its downstream effectors S6 kinase 1 and 4EBP1/eIF4E in a tuberin-dependent manner [8, 9]. Our data from western blot (WB) assays showed PC1-5TMC inhibits phosphorylated S6 (P-S6) and 4EBP1 (P-4EBP1) (Figures 1(e) and 1(f)), which are in line with those of previous reports and supported that the mTOR pathway mediates the regulation of cell proliferation/protein translation by PC1.

### 3.2. PC1-Inhibited Proliferation Depends on PP2A/B56*α*

**Figure 2:**
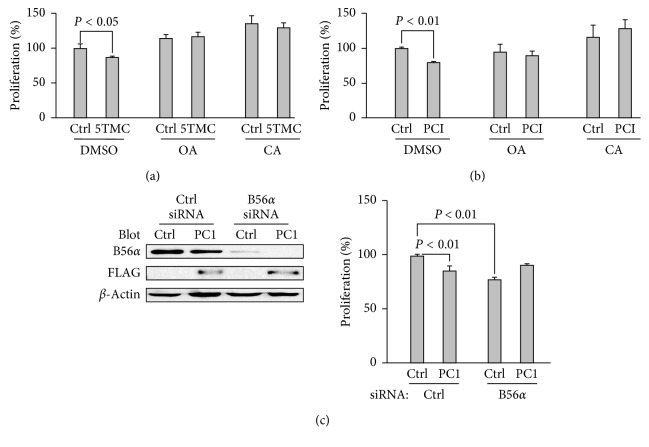
PP2A-dependent regulation of cell proliferation by PC1 in HEK293T cells. (a) Effects of PC1-5TMC on proliferation with treatment of PP2A inhibitors OA or CA (1 nM). Shown are averaged data (*N* = 4). HEK293T cells transiently expressing GFP-PC1-5TMC or GFP were plated in multiple wells of a 96-well plate after 24 hr of transfection and grown for another 24 hr for cell proliferation assay labelling with or without OA or CA treatment. (b) Effects of WT PC1 on proliferation with treatment of OA or CA (1 nM). HEK293T cells stably expressing flag-tagged full-length PC1 after 24 hr of culture replacing the above cells performed as shown in (a) (*N* = 4). (c) Effects of full-length PC1 on proliferation with siRNA knockdown of PP2A/B56*α*. HEK293T cells stably expressing WT PC1 or GFP vector were transiently transfected with B56*α* siRNA and grown for 24 hr before cell proliferation assay. Left panel, effectiveness of siRNA assessed by WB. Right panel, averaged data (*N* = 4).

PP2A was shown to regulate translation initiation through dephosphorylating translational regulators 4EBP1 and p70^s6k^ [16], suggesting that PP2A may act as a mTOR phosphatase to regulate downstream effectors in PC1-inhibited cell growth. To determine whether PC1 inhibits translation and/or proliferation through PP2A, we first examined cell proliferation using PP2A inhibitors OA and CA. After treating cells with 1 nM of OA or CA, which was reported to inhibit the PP2A activity while exhibiting no effect on PP1 [21], we found that PC1-5TMC (Figure 2(a), middle panel) and WT PC1 (Figure 2(b), right panel) no longer exhibit inhibitory effect on proliferation, suggesting that the inhibition of proliferation by PC1 is PP2A-dependent.

PP2A holoenzyme is regulated by its variable regulatory B subunit B56*α* [11]. To determine whether the proliferation regulated by PC1 depends on PP2A-B56*α*, we knocked down B56*α* by siRNA. We found that knockdown of B56*α* abolished the effect of PC1 on proliferation when the expression of B56*α* was completely inhibited (Figure 2(c)), which further supported and verified that the PC1-inhibited cell proliferation is through PP2A (B56*α*).

On the other hand, as shown in the left panel of Figure 2(c), the WB result of the first two bands suggested PC1 reduces the expression of B56*α* without knockdown while that of the third band showed B56*α* was depressed by knockdown and both of the two reduced B56*α* correspond to the decreased cell proliferation shown in the right panel of Figure 2(c), which indicated that PC1-inhibited proliferation could be caused by low expression of B56*α*. Moreover, the further reduction in cell proliferation is expected to be seen in the fourth band when there was no expression of B56*α*; however, cell proliferation was not decreased but increased or it was abolished in this condition, which implied that B56*α* expression or existence is required by PC1-inhibited proliferation.

### 3.3. PC1 Upregulates the PP2A Activity through Decreasing the B56*α* Expression

It was previously thought that overexpression of B56*α* results in an increased level of eIF4E phosphorylation, likely due to decreased PP2A activity [22], and reduced B56*α* expression increases cardiac PP2A activity [23]; that is, nonphosphorylated B56*α* inhibits the activity of purified PP2A [24]. Based on the fact that PC1 upregulates the PP2A activity because PC1 inhibited P-S6 and P-4EBP1 and on the downregulation of the PP2A activity by B56*α*, we deduce that PC1 increases the PP2A activity on substrates through downregulating expression of B56*α*.

**Figure 3:**
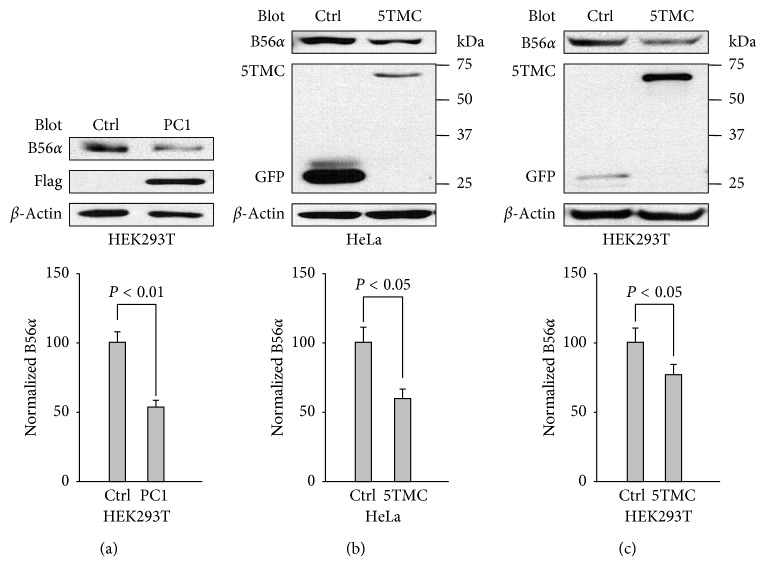
Effects of overexpression of PC1 on B56*α* in cell lines. (a) The upper panel shows effects of WT PC1 on the expression of B56*α*. 100 *μ*g of total protein from HEK293T cells stably expressing full-length PC1 or GFP vector was loaded for WB. The lower panel shows data representing the averaged level of B56*α* after normalization to *β*-actin from the upper panel. Shown is averaged B56*α*/*β*-actin (*N* = 5). *β*-Actin served as a loading control. (b) HeLa cells transiently expressing GFP-PC1-5TMC or GFP were performed as in Figure 3(a) (*N* = 5). (c) HEK293T cells transiently expressing GFP-PC1-5TMC or GFP were performed as in Figure 3(a) (*N* = 5).

Our western blot experiments using HEK293T cells revealed that besides low levels of P-S6 and P-4EBP1 (Figures 1(e) and 1(f)), expression of PC1 results in a decrease in the expression of B56*α* (Figure 3(a)) which actually has been shown in the left panel of Figure 2(c). Similar results were observed in HeLa or HEK293T cells overexpressing PC1-5TMC (Figures 3(b) and 3(c)). The results showed that PC1 downregulates the expression of B56*α*, which indicated that the dependence of PC1-inhibited proliferation on PP2A is mediated by the negative regulation of B56*α* on the PP2A activity.

### 3.4. PC1 Downregulates B56*α* Expression Likely through mTOR

**Figure 4:**
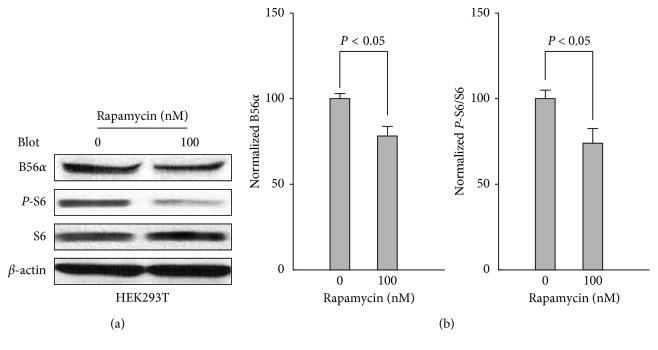
Inhibition of B56*α* expression by rapamycin. (a) Effects of rapamycin (100 nM) on the expression of B56*α*, P-S6 and S6 in HEK293T cells. (b) The left panel shows data representing the averaged level of B56*α* after normalization to *β*-actin from panel a (*N* = 4). The right panel shows averaged and normalized ratios (%) of P-S6/S6 (*N* = 4).

B56*α* expression is regulated via mTOR because the mTOR inhibitor rapamycin blocks B56*α* expression in REH cells [25]. Our WB results also found that rapamycin inhibits both P-S6 and B56*α* in HEK293T cells which further supports that mTOR can promote B56*α* expression (Figure 4).

**Figure 5:**
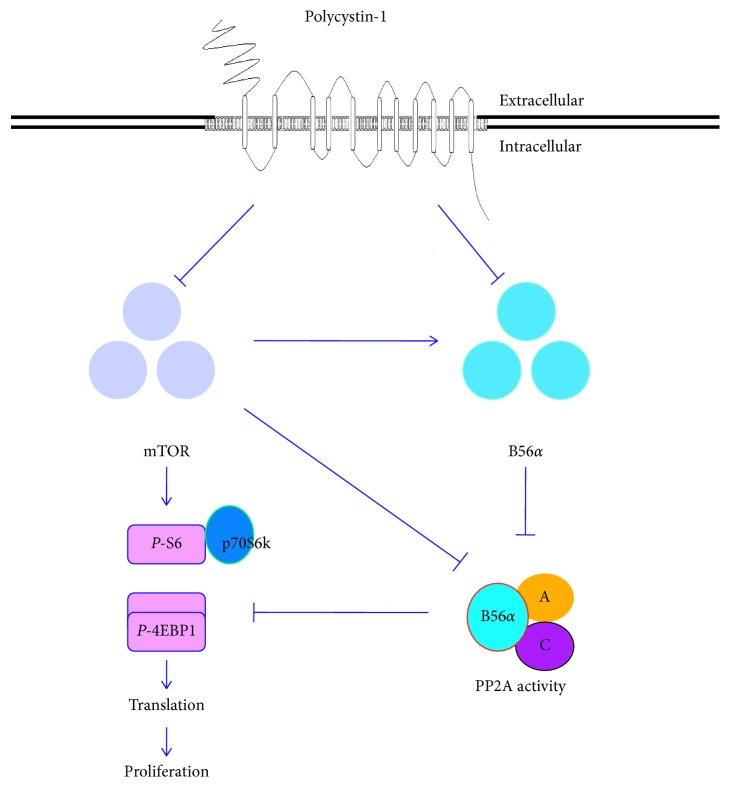
Schematic model for the inhibition of cell proliferation/protein translation by PC1 through PP2A/B56*α*. PC1 inhibits protein translation and cell proliferation by suppressing mTOR and/or decreasing the expression of B56*α* to improve PP2A activity.

Similar to the potency of B56*α* for inhibition of PP2A, several reports have showed that mTOR negatively regulates the PP2A activity [26, 27]; in addition, the previous results have also showed that PC1 downregulates mTOR [9, 28] and that B56*α* inhibits the activity of PP2A [22, 23]. A model can be presented from our present results and those previously reported by others, which is based on the fact that downregulation of B56a and/or mTOR increases the PP2A activity that mediates the inhibition of cell proliferation/translation by PC1 (Figure 5). Therefore, it is highly possible that PC1 negatively regulates expression of B56*α* by negatively controlling mTOR.

## 4. Discussion

The current evidence and research progress showed three main signaling pathways including B-Raf/methyl ethyl ketone (MEK)/extracellular regulated protein kinases (ERK) signaling cascade, mTOR, and nuclear factor of activated T-cell (NFAT) pathways involving increased cell proliferation, fluid secretion, and kidney cyst development seen in ADPKD which may arise due to either the loss of polycystin complex function or an imbalance in the PC1/PC2 ratio [7, 29]. However, PC1 was previously shown to slow cell proliferation and inhibit apoptosis [8, 9, 30]. In our study, the data showed that PC1 inhibits cell proliferation and/or protein translation and dephosphorylates downstream effectors of mTOR. These results were in line with those of previous reports about PC1-controlled cell growth (size) due to the downregulation of mTOR, S6K1, and 4EBP1 [8, 9]. The following results, with the inhibitors of PP2A and knockdown of PP2A/B56*α*, verified that PP2A/B56*α* causes PC1-inhibited cell proliferation. Furthermore, we first found PC1 downregulates expression of B56*α*. For decreased levels of S6 and 4EBP1 phosphorylation due to increased PP2A activity caused by either reduced B56*α* or PC1 [9, 22, 23], the dependence of PC1-inhibited proliferation on PP2A can be, at least partly, explained as low expression of B56*α*.

Both B56*α* and mTOR negatively regulate the PP2A activity [22–24, 26, 27]. Our result also showed the inhibitor of mTOR rapamycin suppresses B56*α* expression, which indicated that PP2A is a major phosphatase of mTOR to regulate downstream effectors by B56*α*. Furthermore, previous WB results have showed that PC1 upregulates P-AKT [30] which indicates that B56*α* expression regulated by PC1 is independent of AKT, although experiments showed that inhibition of AKT also suppresses B56*α* expression in acute lymphoblastic leukemia-derived REH cells [25].

Also, Ruvolo et al. considered that although mTOR kinase seems to be involved in rapamycin-inhibited B56*α* expression, it is unlikely that mTOR regulates B56*α* directly by such kinase pathways, because expression of B56*α* can be restored with a proteasome inhibitor [25]. They also found that protein kinase R (PKR) can protect B56*α* by suppressing proteasome-mediated proteolysis [25]. Therefore, we speculate the possibility that mTOR supports B56*α* expression indirectly by promoting PKR that directly protects the B subunit from proteolysis.

B56*α* itself can also be regulated by reversible phosphorylation and dephosphorylation. Beside PKR, protein kinase A (PKA) [31], cyclin-dependent kinase (CDK), protein kinase C alpha (PKC*α*), checkpoint kinase (CHK), and PP2A itself can regulate the phosphorylation of B56*α* to promote or suppress B56*α* function [22, 32–34]. Moreover, the phosphorylation of B56*α* by PKA or PKR increased the PP2A activity, but conversely, the potency of B56*α* for PP2A inhibition was markedly increased by a phosphorylation of B56*α* at Ser41 by PKC [32]. Therefore, more experiments will also be needed to determine the relationship between B56*α* phosphorylation and PP2A activity or mTOR in the inhibition of proliferation and translation by PC1.

## 5. Conclusions

In this study, we first demonstrated that PC1-inhibited proliferation depends on PP2A/B56*α*, which can be accounted for by the expression of B56*α*. The current and reported results reveal that PC1 is most likely to downregulate B56*α* expression through mTOR. Further experiments will explore the involvement of mTOR-dependent B56*α* expression and B56*α* phosphorylation in PC1-inhibited proliferation/translation.

## Data Availability

The data used to support the findings of this study are available from the corresponding author upon request.
